# Bus Single-Trip Time Prediction Based on Ensemble Learning

**DOI:** 10.1155/2022/6831167

**Published:** 2022-08-11

**Authors:** Haifeng Huang, Lei Huang, Rongjia Song, Feng Jiao, Tao Ai

**Affiliations:** ^1^Department of Information Management, School of Economics and Management, Beijing Jiaotong University, Beijing 100044, China; ^2^Department of Information Management, School of Management, Hangzhou Dianzi University, Hangzhou 310018, China

## Abstract

The prediction of bus single-trip time is essential for passenger travel decision-making and bus scheduling. Since many factors could influence bus operations, the accurate prediction of the bus single-trip time faces a great challenge. Moreover, bus single-trip time has obvious nonlinear and seasonal characteristics. Hence, in order to improve the accuracy of bus single-trip time prediction, five prediction algorithms including LSTM (Long Short-term Memory), LR (Linear Regression), KNN (K-Nearest Neighbor), XGBoost (Extreme Gradient Boosting), and GRU (Gate Recurrent Unit) are used and examined as the base models, and three ensemble models are further constructed by using various ensemble methods including Random Forest (bagging), AdaBoost (boosting), and Linear Regression (stacking). A data-driven bus single-trip time prediction framework is then proposed, which consists of three phases including traffic data analysis, feature extraction, and ensemble model prediction. Finally, the data features and the proposed ensembled models are analyzed using real-world datasets that are collected from the Beijing Transportation Operations Coordination Center (TOCC). Through comparing the predicting results, the following conclusions are drawn: (1) the accuracy of predicting by using the three ensemble models constructed is better than the corresponding prediction results by using the five sub-models; (2) the Random Forest ensemble model constructed based on the bagging method has the best prediction accuracy among the three ensemble models; and (3) in terms of the five sub-models, the prediction accuracy of LR is better than that of the other four models.

## 1. Introduction

Rapid economic growth is accompanied by increasingly serious traffic congestion on urban roads. The vigorous development of public transportation is considered as one of the effective means to alleviate traffic congestion. Many factors are considered as relevant to the acquisition of dynamic public transport information and the prediction of bus single-trip time, including the decision-making of passenger bus travel, the priority control of bus vehicles at intersections, and the intelligent scheduling and the real-time information dissemination of public transport [1]. Furthermore, the accurate prediction of bus single-trip time is the prerequisite and basis for achieving the bus priority and the intelligent scheduling, which could help to effectively reduce passenger waiting time and improve passenger satisfaction, in order to further greatly impact the bus service level and bus travel attractiveness. More specifically, the role of accurate prediction of bus single-trip time is mainly reflected in the following aspects: providing real-time information such as bus arrival time for passengers, efficient management of bus fleets, improving bus service quality, and providing bus priority signals and information for traffic managers. Therefore, it is of great significance to explore the prediction method of bus single-trip time and improve the prediction accuracy of the bus running time, which could help to improve the attractiveness of public transport and promote the development of public transport [2].

The prediction of bus single-trip time has attracted extensive attention in the past decade or so, and various predicting methods have been proposed in the literature [3]. Among them, typical methods are mainly statistical models, such as Auto Regressive Integrated Moving Average (ARIMA) models [4], Seasonal Auto Regressive Integrated Moving Average (SARIMA) model [5], the Grey Model (GM) [6], Kalman filtering [7], and spectral analysis. Although the above statistical methods perform well in capturing linear relationships, they have limited ability to capture nonlinear features [8]. In recent years, many scholars have also turned to some nonlinear methods, such as Support Vector Regression (SVR) [9] and Artificial Neural Networks (ANNs) [10]. Moreover, popular ANN models in this field include Back Propagation Neural Networks (BPNNs) [11], Extreme Learning Machines (ELMs) [12], and Deep Belief Networks (DBNs).

Bus operation could be affected by many random factors (e.g., weather, traffic congestion, and passenger flow change), which makes it very difficult to predict the running time of a bus accurately. In terms of predicting the bus running time by using a single prediction model, the most popular methods include time series models, artificial neural networks, and Kalman filtering. (1) Time series models mainly rely on the similarity between future information and historical information, and when the average situation of historical data changes, it would lead to an obvious deviation of the prediction results. Moreover, time series models also have significant lags when making real-time prediction [13]. (2) Kalman filtering technique, which is formed by introducing the state space into modern control theory, has been applied to short-term traffic demand and travel time prediction of highways [14]. Since the Kalman can fully adapt to irregular changes (Kalman gain), it is more suitable for single-step predicting, but its prediction accuracy decreases significantly in multi-step predicting [15]. (3) Artificial neural network is a model that explores the nervous system functions of the human brain by modeling and linking neurons (i.e., the basic units of the human brain) to simulate the functions of the human brain. The principle is an artificial system with intelligent information processing functions such as learning, association, memory, and pattern recognition. Its unique nonlinear adaptive information processing capability makes it an effective way to solve complex combinatorial optimization problems [16]. However, the generalization ability of neural network algorithms is limited due to the structure determination, over- and under-learning, and local convergence problems of neural network algorithms [17]. Among the existing prediction methods, the multiple regression model has poor applicability and low predicting accuracy. Neural network models can fit nonlinear systems well, but a large historical dataset need to be trained before use, which also has limit performance on under-learning and over-learning, as well as local optimality. Support Vector Machine (SVM) has strong learning ability and fault tolerance ability, and its generalization ability is better than neural network. However, similar to neural network models, pretraining is also needed for SVM, which makes it difficult to be used for real-time prediction. The Kalman filter model is applicable for predicting online and perform well, but it is difficult to guarantee the accuracy of nonlinear and non-Gaussian state models. To sum up, in the area of bus single-trip time prediction, each single method has advantages and disadvantages. In order to further improve the prediction accuracy, this study adopts the idea of ensemble learning and concentrates the prediction advantages of several single prediction models by building an ensemble model.

Ensemble learning is a widely used approach in prediction using ensemble predictive models in machine learning. It is based on the principle of integrating different sets of learners for improving prediction accuracy [18]. The dominant area of research by scholars is currently designing ensemble models that enhance weak learners to strong learners and ensemble multiple learners generated by the same algorithm [18]. In ensemble learning, the prediction accuracy is greatly improved by combining multiple learners and the ensemble model performs better than each sub-model. This result is due to the diversity among sub-models, which reduces the risk of using isolated models and meanwhile compensates the weakness of sub-models. In addition, ensemble models can solve many problems that individual models cannot solve. For instance, the transit running time of urban public transportation is dynamic and stochastic. It is difficult for a single model to fit its trend well, and ensemble learning can better compensate for this deficiency. Bus single-trip time prediction has attracted much attention in the recent years, and some major challenges about bus single-trip time prediction have been pointed out as follows [19]:

1. It is necessary to predict the bus single-trip time based on dynamic spatiotemporal and weather conditions.
2. Further exploration of ensemble learning in bus single-trip time prediction is needed.
3. It is necessary to provide a highly universal bus single-trip time prediction method for public transport managers and passengers.

Toward meeting the above challenges, this study focuses on predicting the bus single-trip time using ensemble models. Based on the validation analysis using real-world data, we compare and analyze the prediction effects of five sub-models including LSTM (Long short-term memory), LR (Linear Regression), KNN (K-Nearest Neighbor), XGBoost (Extreme Gradient Boosting), and GRU (Gate Recurrent Unit). The ensemble learning models are constructed by using three ensemble methods, and a data-driven prediction framework is further proposed. The main contributions of this paper are summarized as follows:

1. A data-driven predicting framework is proposed for bus runtime prediction, which consists of three phases: (a) data analysis-preparing bus runtime data from TOCC; (b) feature extraction-extracting key features for forecasting based on bus runtime time series and external data; (c) feature extraction-extracting key features required for predicting based on bus runtime time series and external data; and (d) prediction modeling-constructing three ensemble models for predicting.
2. The ensemble learning method was introduced into the short-term transit runtime prediction model based on the three-model ensemble methods including bagging (Random Forest), boosting (AdaBoost), and stacking (Linear Regression).
3. Based on the real-world bus single-trip time data, after case analysis, the prediction results of LSTM, LR, KNN, XGBoost, and GRU and three ensemble models are compared and analyzed, and the advantages and disadvantages of these prediction models in bus single-trip time prediction are summarized.

This study significantly improves the bus single-trip time prediction accuracy by ensemble learning and provides a new modeling method for quantitative bus research, significant for theoretical guidance and methodological innovation.

This paper is organized as follows.  Section 1 is the introduction, which mainly introduces the research background, motivation, and contributions.  Section 2 is the literature review, which mainly sorts out the existing methods in the field of bus single-trip time prediction and points out the defects and deficiencies in current research. In Section 3, the methodology of this paper is proposed, which involves three ensemble models constructed based on five single prediction algorithms by using three-model ensemble methods. Furthermore, the case analysis is presented in Section 4, which includes real-world data description, experimental procedures by employing the methodology, and comparative analysis of prediction results. Then, the discussion comes in Section 5; according to the analysis results, the advantages and disadvantages of the three ensemble models and five single models are summarized. And the capability of applicability and generalization of the proposed methodology is discussed and elaborated. Finally, the paper is concluded in Section 5; the future perspectives are set as well.

### 1.1. Related Works

So far, scholars have proposed various operational and predictive models of public transportation. Popular models include regression models, time series (TS) models, ANN models, SVM models, traffic simulation and dynamic traffic simulation, and dynamic traffic assignment models [20]. Ghosh et al. [21] considered the effect of data fluctuations in the TS and established an autoregressive moving average TS model to predict the vehicle running time by data fitting and residual analysis. However, the large white noise in the data residual series negatively affects the prediction accuracy, indicating that the model lacks the treatment of the complexity and variability of urban traffic (Gu et al. [22]). A nonlinear regression model was developed considering factors such as road section length, traffic density, number of bus stops, and the vehicle turns. Agafonov and Yumaganov [23] developed a multiple linear regression model and an ANN model based on bus operation data in Samara and Russia by using real-time dynamic traffic data and historical data of bus stopping time. In the analysis of the results, it was found that the ANN model possessed higher prediction accuracy and lower prediction error. Yu et al. [24] improved the SVM algorithm by introducing a decay factor to reduce the prediction error through the decay factor dynamically. Bie et al. [25] performed bus single-trip time prediction based on bus GPS data, but the model built did not consider the real-time status of traffic flow and roads. Dhivya Bharathi et al. [26] used the historical data averaging method and TS method for predicting the transit section running time but lacked influencing factors. Chang et al. [27] predicted the bus single-trip time using a regression algorithm based on historical data well, but the predictive model is quite complicated. Since bus single-trip time is greatly influenced by dynamic traffic conditions, Liu et al. [28] used a particle filtering algorithm to predict the bus arrival time with the nonlinear and non-Gaussian characteristics in real-time. Hua et al. [29] proposed a bus journey time prediction method based on SVR and interval upper and lower bound estimation methods, which considered the uncertain factors in the bus operation. However, the method only turned the point prediction values into prediction intervals and did not study the road traffic status as a variable. Wu S. proposed the Adaptive Boosting (AdaBoost) algorithm [30]. The Bagging algorithm uses sequential sampling with high operational efficiency and practical applications. Bagging algorithm uses self-sampling combined with a base learner, which was improved to Random Forest (RF) in 2001 [31]. Wolpert proposed the stacked generalization model, but the stacking algorithm only provides the ensemble idea, and the choice of its base learner is somehow subjective [31].

From above, it is known that there are mainly predicting methods based on statistics, intelligent algorithms, and combined models for bus single-trip time prediction. Based on the current mainstream algorithms in classification, this study divides three major categories into six subcategories, among which the statistical methods include historical contemporaneous and time series; intelligent algorithms include machine learning and deep learning; combinatorial models include ensembled models and combined models. More specifically, the advantages and disadvantages of the commonly used models in each category are compared in Table 1.

Although single models are studied by many researchers and proved to be suitable for many cases, some shortcomings still exist. For example, in the study of TS, Billings and Yang [51] used the ARIMA model to predict the arterial travel time with the GPS data collected from Minnesota State Highway 194. ARIMA is a very simple time series forecasting method. It can only capture linear relationships in nature, but its capture of nonlinear relationships is not accurate. Moreover, ARIMA requires that the time series data are stationary or are stable after differencing (mean and variance are stable). For the bus single-trip time of this study, under the influence of various factors, the mean and variance of the data will change greatly even after the nonlinear data are differentiated. Therefore, ARIMA is not selected as a sub-model in this paper. The bus travel time was also predicted using the SARIMA model in [5]. However, these models have not considered the dynamic factors that affect bus travel times, such as the traffic condition. The Kalman filter models can obtain accurate prediction results with limited historical data; however, the accuracy decreases with increasing time steps [52]. Moreover, the performance of the Kalman filter models becomes unstable if there is big difference in the predicted value between two consecutive time windows [53]. The regression model requires a large amount of historical data for training to achieve the expected prediction accuracy [54]. Although machine learning and deep learning methods are also quite popular at present, their low training efficiency and interpretability make them less utilized in the study of bus single-trip time [19, 55]. Alternatively, it is a much better way to fuse the results from different predictive methods. Combined models could achieve better accuracy compared to each single predictor. The ensemble learning models have been proved to be able to achieve much better performance in prediction accuracy than individual ones. Nowadays, ensemble learning has been used in many fields of traffic prediction, such as traffic sign detection and recognition [56], traffic speed [52], short-term traffic volume [57], and traffic incident detection [58]. The advantage of ensemble learning is that the benefits of multiple learners can be integrated to improve the accuracy of predictions. The general approach is to generate multiple individual learners first and then ensemble them for predicting using specific ensemble strategy [59]. A type of ensemble is called “homogeneous” if the individual learners are of the same type and “heterogeneous” if else. Individual learners need to be accurate and diverse [60, 61].

More specifically, the advantages of ensemble learning are as follows. Overall, ensemble learning has a high accuracy rate and good resistance to noise, which makes the model less prone to overfit due to the introduction of randomness [59]. It is insensitive to outliers, so it can handle very high dimensional data without the necessity to select features. Ensemble models can handle both discrete and continuous data, and in addition, the dataset does not need to be normalized [62]. Meanwhile, the overall training speed is relatively impressive. However, current ensemble methods are not explicitly designed for dealing with spatiotemporal data. Therefore, how to effectively ensemble multiple models while utilizing the spatiotemporal information remains a challenging, especially for practical problem in the real world. In addition, a few studies focus on using ensemble learning methods to predict bus single-trip time [63].

In summary, each single predictive model has certain advantages and disadvantages. Therefore, in this paper, three ensemble learning methods including Random Forest, AdaBoost, and Linear Regression are used to fuse the prediction results of sub-models. The prediction results of the ensemble models could increase the prediction stability even if there are quite large deviations among the prediction results of the sub-models.

### 1.2. Methodology

#### 1.2.1. Ensemble Model Construction of RF-Bagging

Bagging is a method for learning multiple weak learners independently. Bagging is known as bootstrap aggregating. Bootstrap randomly selects *n* training samples from *n* training samples, which allows the generation of a repeated set of samples that are slightly different from the original training set.

**Figure 1:**
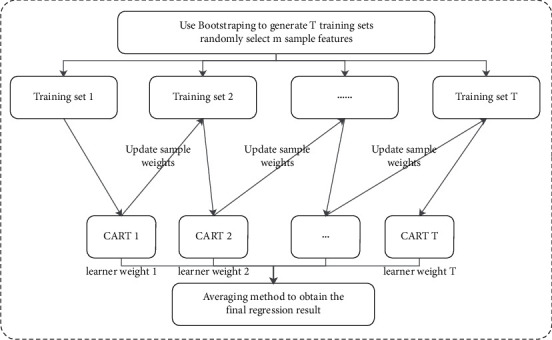
Structure of the ensemble model based on Random Forest.

Random Forest (RF) is an evolved version of the Bagging algorithm; the idea is still bagging but with unique improvements [64]. First, Random Forest uses the CART decision tree as a weak learner. Secondly, Random Forest improves the decision tree building based on the use of decision trees. For a normal decision tree, we choose an optimal feature among all the *n* sample features on the node to do the left and right subtree divisions of the decision tree. But RF selects a part of the sample features on the nodes by randomly selecting a number less than *n*, which is assumed to be *n*_*sub*_ And then, among these randomly selected *n*_*sub*_ Then, among these randomly selected sample features, an optimal feature is selected to do the left and right subtree partitioning of the decision tree. This further enhances the generalization ability of the model. If *n*_*sub*_=*n* , then there is no difference between the CART decision tree of RF and the ordinary CART decision tree at this time. *n*_*sub*_ The smaller the model is, the more robust it is, but of course the fit to the training set become worse. In other words, *n*_*sub*_, the smaller the model, the smaller the variance of the model will be reduced, but the bias will be increased. A suitable value is usually obtained by cross-validating the tuning parameters in practical cases. *n*_*sub*_ value of the model. The structure of the ensemble model based on the random forest is shown in Figure 1.

1. The input is a sample set of *D*={*x*_1_, *y*_1_, (*x*_2_, *y*_2_),…, (*x*_*m*_, *y*_*m*_)}, and the number of weak classifier iterations is *T*.
2. For *t*=1,2,…, *T*: randomly sample the training set for the *t* th time, randomly select *m* features for each training set, and obtain a sampling set *𝒟*_*bs*_ containing *m* feature samples.
3. Using the sampling set *𝒟*_*bs*_ to train the *t* th decision tree model *G*_*t*_(*x*), when training the nodes of the decision tree model, select a portion of sample features among all the sample features on the node, and choose an optimal feature among these randomly selected partial sample features for the left and right subtree partitioning of the decision tree.
4. The regression algorithm is used, and the value obtained by arithmetic averaging the regression results obtained from *T* weak learners is the final model output.

The entire algorithm flow is described in Algorithm 1.

Using Random Forest algorithm for model ensemble has the following advantages: the training can be highly parallelized, which is advantageous for the training speed of large samples in the era of big data; the introduction of two randomness makes Random Forest have good anti-noise ability and is insensitive to partial feature missing; due to the use of random sampling, the variance of the trained model is small, and the generalization ability is strong. Compared with Adaboost and GBDT based on Boosting, the Random Forest algorithm is relatively simple to implement; since the decision tree nodes can be randomly selected to divide the features so that the model can still be trained efficiently when the sample features are of high dimensionality; wrong predictions are made only when more than half of the base learners are in error: Random Forest is very stable, even if a new data point appears in the dataset, the whole algorithm will not be affected too much, it will only affect one decision tree, and it is not easy to affect all decision trees.

However, features with more value divisions are likely to have a greater impact on RF decision-making, thereby affecting the effect of the model.

#### 1.2.2. Ensemble Model Construction of AdaBoost-Boosting

Boosting is a class of algorithms that boosts weak learners to strong learners. It is a serial idea where serialization is performed [61]. The basic idea is that increasing the weights of the samples that the previous base learner incorrectly predicted makes the subsequent base learners pay more attention to these mislabeled samples and correct these errors as much as possible. Until *T* base learners are trained, eventually, these *T* base learners are weighted and combined.

**Figure 2:**
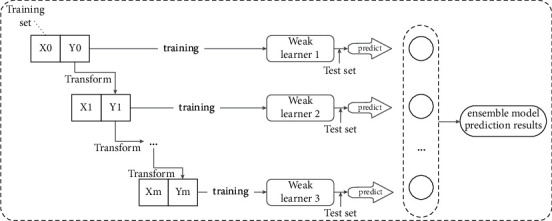
Structure of AdaBoost-based ensemble model.

As shown in Figure 2, boosting adaptively fits multiple base learners in sequence; the current model training is based on the training results of the previous base learner, and the current base learner increases the weight of the misestimated samples, which in turn reduces the prediction error rate. Therefore, unlike bagging, boosting mainly focuses on reducing the bias of the model, and usually, the base model is chosen to have high bias and low variance. If a decision tree is chosen as the base model, most decision trees with shallow depth are selected, reducing the computational cost of model fitting.

The loss function used by AdaBoost is the exponential loss function, so the weights and sample distribution of AdaBoost revolve around minimizing the exponential loss function. This study defines the ensemble learner as a linear weighting of the base learner, where *α* is the weight of the base learner:

(1) $$\begin{array}{c} H\left(x\right)=\sum_{t=1}^{T}\alpha_{t}h_{t}\left(x\right). \end{array}$$

Also, the exponential loss function defined by AdaBoost is

(2) $$\begin{array}{c} loss_{\exp}\left(h\right)={Ε}_{x∼D},y\left[e^{-yh\left(x\right)}\right]. \end{array}$$

The specific AdaBoosting iteration, in three steps.

1. Initialize the weight distribution of the training data. If there are N samples, at first all samples are given the same weights: 1/N.
2. Training the weak learner. In training, if a sample point has been learned accurately, the weight of that sample point decreases in constructing the next training set; conversely, the weight of sample points that failed to be learned accurately increases.
3. The weak learners obtained from each training are formed into strong learners. After each weak learner is trained, the weight of the weak learner with a small prediction error rate is increased (i.e., the base model that performs well and plays a larger role). The final linear combination of weak learners is formed.

The entire algorithm flow is described in Algorithm 2.

The advantage of the AdaBoost ensemble model is that the base learner can be constructed using various methods. Simple weak learners can be used without filtering the features and without overfitting. In addition, the AdaBoost ensemble model does not need to know the upper limit of the error rate of the weak learners in advance, and the accuracy of the final strong learner obtained depends on the accuracy of all the weak learners, which allows for digging deeper into the ability of the learners. AdaBoost can adjust the assumed error rate adaptively based on the feedback from the weak learners and performs efficiently.

However, it should be noted that boosting is very sensitive to the noise of the training data because the AdaBoost ensemble model focuses its attention on the error samples. If the training data contain a lot of noisy data, then the base learners will all focus on the noisy data for training, which will instead affect the effectiveness of the whole model.

#### 1.2.3. Ensemble Model Construction of LR-Stacking

The model ensemble idea of stacking is completely different from bagging and boosting. Stacking aggregates base models using a model fusion approach, while bagging and boosting use specified strategies [65]. The idea of the stacking framework is to select heterogeneous base learners to be trained on the training set in parallel. All the trained base models are predicted on the prediction set, and the predicted values of all base models are trained as the training set of another fusion model. When a new dataset is an input, it is first predicted by the base model and then input to the fusion model for the final prediction output. For example, we fit a stacking ensemble model consisting of *m* base learners. First, we divide the training data into the training set and test set, train *m* base learners on the training set, use the trained base learners to predict the prediction set to output *m* predictors, and *m* predictors are used as the training set of the fusion model to train the fusion model. We usually use K-fold cross-validation for model training in practical applications, and logistic regression is generally chosen for the fusion model.

**Figure 3:**
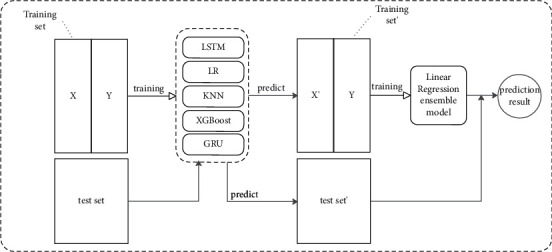
Structure of ensemble model based on Linear Regression.

As shown in Figure 3, based on the idea of stacking, a sub-base model for combining each other base model is trained. This is done by dividing the data into two parts, using one part to train five base models, using the other part of the data to test these base models, and using the output of the five base models as input to train the ensemble model. Instead of organizing the prediction results of the base models, this ensemble of models organizes the models. Theoretically, stacking can organize any model.

The Linear Regression ensemble model is constructed in four steps as follows:

1. Prepare the training set and test set by dividing the training set into five parts: train1, train2, train3, train4, train5.
2. Selected base models. Here, the five base models, LSTM, LR, KNN, XGBoost, and GRU, are selected as the base models. For example, in the XGBoost model part: train1, train2, train3, train4, and train5 are used as validation sets in turn, and the remaining four copies are used as training sets for 5-fold cross-validation for model training; then, the prediction is performed on the test set. This will result in five copies of predictions trained by the XGBoost model on the training set and one copy of predictions on the test set. The five copies are overlapped vertically and combined to obtain the training set for the LR model. The same is done for the rest of the base model.
3. After the five base models are trained, the predicted values of the five models on the training set are used as five “features” for training using the LR model.
4. Using the trained LR model, the final prediction is made based on the five “feature” values constructed from the previous predictions of the five base models on the test set.

The entire algorithm flow is described in Algorithm 3.

The stacking framework is ensemble with different algorithms to make full use of different algorithms to make different observations on data from the different data space perspectives and data structure perspectives to take advantage of the strengths and weaknesses to optimize the results. Therefore, in addition to the same model with different parameters, the base model can also be of different kinds of base models, so this paper selects LSTM, LR, XGBoost, KNN, and GRU, which have their own characteristics. By aggregating different kinds of base models, we can fully learn the changing law of bus single-trip time, and the ensemble results will be more robust and accurate.

#### 1.2.4. Selection of Sub-models

In this paper, the commonly used bus single-trip time prediction models are divided into types, and their advantages and disadvantages are compared and analyzed. Since the sub-models are selected to be as different as possible in terms of internal principles, the selection of sub-models is carried out in this paper from the broad category of model division. Five sub-models were selected from the broad categories of statistics, deep learning, machine learning, and ensemble learning. They are Multiple Linear Regression (LR), K-Nearest Neighbor (KNN), XGBoost, Long Short-Term Memory Network (LSTM), and Gate Recurrent Unit (GRU), and the internal structure and algorithmic principles of each model are briefly described below.

**Figure 4:**
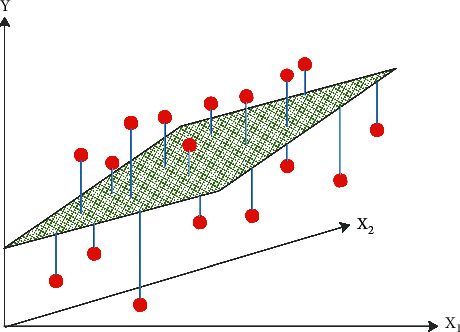
MLR schematic diagram.

MLR: although ARIMA is one of the most commonly used time series models, it is more applicable to scenarios with single factor inputs [66]. The model principle is simple, as shown in Figure 4. The error between the predicted and true values is calculated, and a line (plane or hyperplane) is finally fitted by continuously seeking the optimal solution of the parameters utilizing gradient descent. The linear regression model can capture well the linear patterns present in the data but is insensitive to nonlinear patterns. MLR is very suitable when the dependent variable is affected by two or more features, and there is a linear relationship between multiple independent variables and the dependent variable. In this study, holidays, rainfall, visibility, and temperature are the data characteristics that affect the bus single-trip time, and they are independent and continuous, so multiple linear regression is selected as the sub-model.

**Figure 5:**
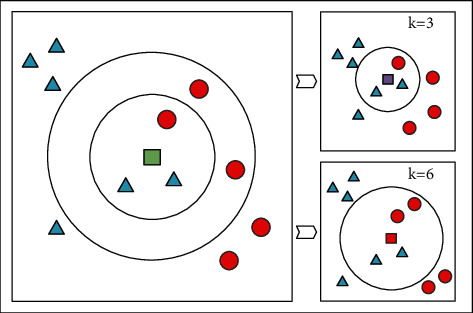
KNN algorithm principle.

KNN: KNN is a distance-based model, and the principle of its classification model algorithm is given in Figure 5. Based on the K-value, the nearby elements are selected and grouped into the class with the highest number of nearby element categories, the most similar class. The regression model is similar, based on the K-value size to select the nearby data and further processing to obtain the predicted value, which is usually processed by directly finding the mean value [67]. This study chooses KNN as the sub-model for the following two reasons. First of all, in terms of model, the principle and implementation of KNN are relatively simple. KNN does not need to estimate parameters and is suitable for dealing with regression problems. Secondly, in terms of data, KNN is not sensitive to the outliers of the data, and has good Lupin performance for the nonlinear data with large noise value such as the bus single-trip time.

**Figure 6:**
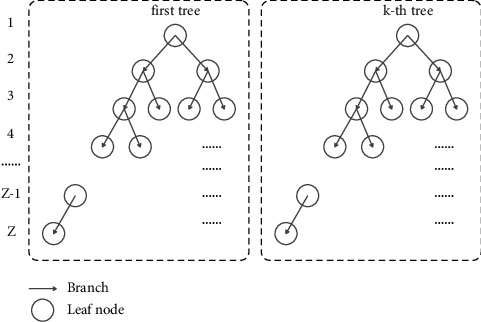
XGBoost principle.

XGBoost: XGBoost is a boosted tree model based on an ensemble learning boosting approach to regression tree as the base model [68]. It has an objective function. The objective function depends only on the first- and second-order derivatives of the data in the error function. In terms of implementation, it can be parallelized (sparse-aware algorithm is proposed) to speed up the training speed; a regular term is added to the objective function, which controls the complexity of the model and helps prevent overfitting; XGBoost supports columns sampling, can reduce overfitting, and reduce computation; it can handle missing values. Based on these advantages, this study selects XGBoost as a sub-model. The principle of the algorithm is shown in Figure 6.

**Figure 7:**
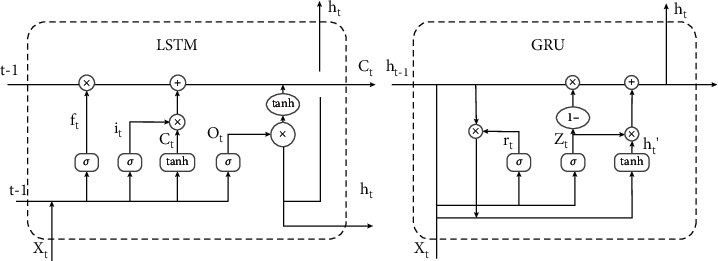
Comparison of the internal structure of LSTM and GRU.

LSTM: long short-term memory (LSTM) is a special RNN, mainly to solve the problem of gradient disappearance and gradient explosion during long sequence training. Simply put, LSTM can perform better in longer sequences than ordinary RNNs. As a deep learning model, CNN is not completely suitable for learning time series, so various auxiliary processing is required, and the effect is not necessarily good. For problems that are sensitive to time series, LSTMs are usually more suitable. LSTM is an excellent variant model of RNN. It inherits the characteristics of most RNN models and solves the vanishing gradient problem caused by the gradual reduction of the gradient backpropagation process. Therefore, LSTM is very suitable for dealing with time series [69]. So, this study chooses LSTM as one of the sub-models. The principle of LSTM is shown in Figure 7.

GRU: GRU is a very effective variant of the LSTM network, which is simpler and more effective than the structure of the LSTM network, so it is also a very streamlined network at present [70]. Three gate functions are introduced in LSTM: input gate, forget gate, and output gate to control the input value, memory value, and output value [71]. Moreover, there are only two gates in the GRU model: update gate and reset gate. The number of parameters of LSTM is 4 times that of RNN. If the number of parameters is too large, there is a risk of overfitting. GRU only uses two gated switches, which achieves results close to LSTM. In order to avoid overfitting of LSTM, GRU was selected as one of the sub-models for comparison in this study. The specific structure is shown in Figure 7.

#### 1.2.5. Model Construction and Prediction Steps

**Figure 8:**
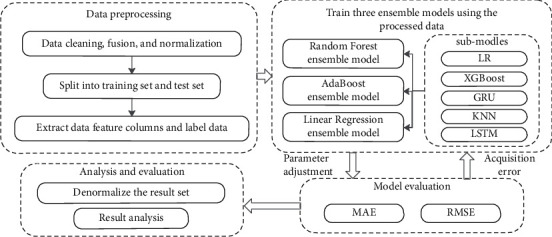
Prediction steps.

The specific prediction steps are shown in Figure 8. Firstly, the fused processed data are loaded into the dataframe, and the data are split in the ratio of 6 : 1 : 1 before feeding them into the model and divided into the training data set, the test data set, and the prediction data set.

Feature selection of the data is performed afterward to select the key features used to predict the target data. In this study, feature selection is performed using the method of removing low variance features to ensure that the complexity associated with redundant features is reduced while maximizing feature relevance. This step identifies the most meaningful subset of features by removing low variance features, including date, bus route up and down, whether a holiday, temperature, visibility, and precipitation.

Next, the organized training data set is input to each of the five sub-models for model training, and the model is trained to find the most suitable weights and optimal model parameters to minimize the error between predicted and actual values. The parameters in each algorithm are determined by the grid search method before using them for prediction.

The training dataset is fed into the ensemble model consisting of sub-models that have been tuned with hyperparameters to train the ensemble model separately, and the parameters of the ensemble model are tuned.

For RF-Bagging, firstly, a fixed number of samples are collected from the original training set by random sampling (bootstrap), but after each collection, the collected samples will be put back. Randomly collecting the same number of samples as the number of training samples *m* can make the number of samples in the sampling set and training set the same, but the sample content is different. Secondly, based on the gradient boosting tree, RF improves the establishment of the decision tree. We will select an optimal feature from all the *n* sample features on the node to divide the left and right subtrees of the decision tree. Finally, because we are studying the regression problem, we arithmetically average the obtained regression results to obtain the final model output.

For AdaBoost-Boosting, the training set of each round is unchanged, but the weight of each example in the training set in the classifier changes. The weights are adjusted according to the classification results of the previous round. The weights of the samples are continuously adjusted according to the error rate. The larger the error rate, the greater the weight. Individual prediction functions can only be generated sequentially, because the results of the previous model round are required for the latter model parameters.

For LR-Stacking, this stacked ensemble model is relatively simple, and the prediction results of the five sub-models are used as the data of the secondary learner, that is, as the training set of the LR model. The prediction result of the trained LR model is the prediction result of the ensemble model.

Model validation is performed using a test dataset after the model is trained. The same parameters used for training the model are used for data validation, and the same feature values are selected on the estimated validation data used during model training. This step aims to verify the prediction accuracy of the model and minimize the output error of the validation data. Model parameters are tuned based on training and validation results to find the parameters that apply to the test data based on the features of different sub-models.

Finally, the prediction accuracy of the ensemble model is then validated using test data.

## 2. Case Analysis

### 2.1. Description of Usage Data

**Figure 9:**
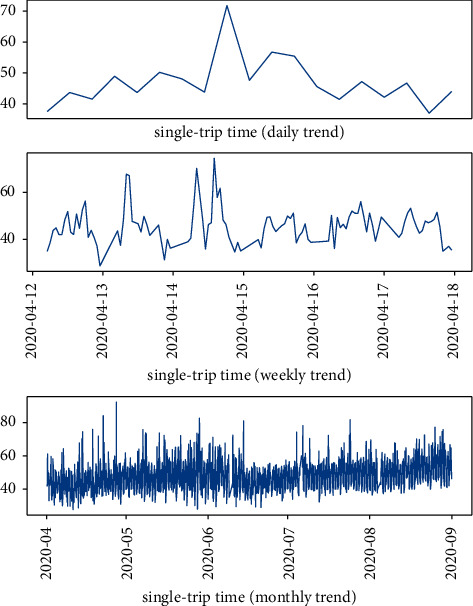
Bus single-trip time data trend graph (daily, weekly, monthly).

The data used in this study are the bus single-trip time data of Beijing 2 from April 1, 2020, to August 31, 2020, under the normal scenario, with a total of 3512600 entries. As shown in Figure 9, it can be seen from the figure that the data have obvious seasonal characteristics of time series. The unit of the *y*-axis in the figure is minutes.

**Figure 10:**
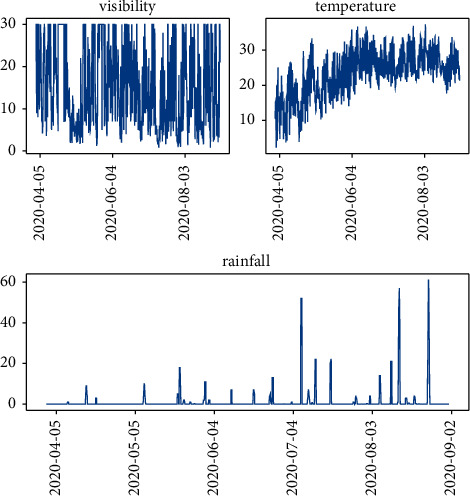
Weather data trend graph.

Holiday and Beijing weather data in the same period as the bus single-trip time data are 1210 items with a time granularity of 3 hours. The temperature unit is Celsius, the visibility unit is a kilometer, and the precipitation unit is millimeters. The data analysis graph is shown in Figure 10.

**Figure 11:**
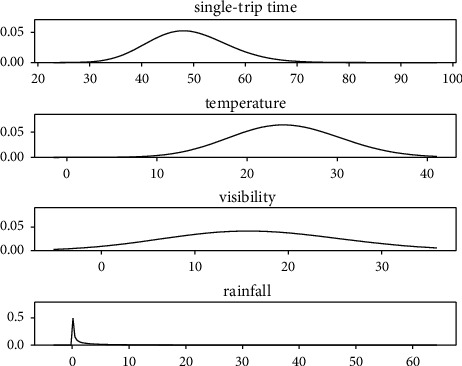
Data distribution.

**Figure 12:**
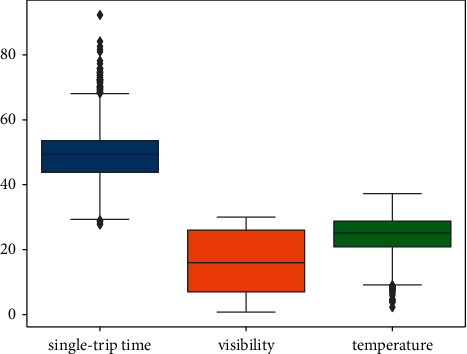
Weather data box plot.

As shown in Figure 11 and Figure 12, the bus single-trip time running time and weather data are combined to see that the data of bus single-trip time are mainly concentrated in the interval of 4 0 minutes to 60 minutes. The values of temperature are mainly distributed between 20 and 30. The distribution of visibility and rainfall is more scattered, and the values change more randomly.

**Figure 13:**
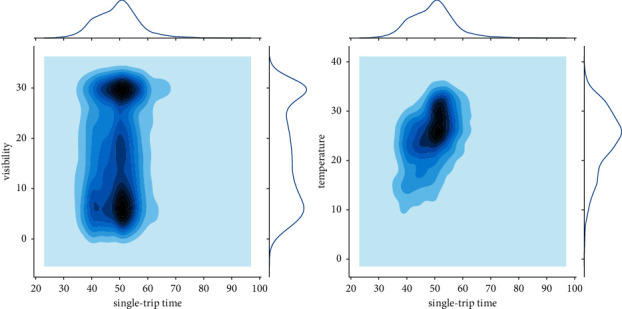
KDE plots (single-trip time-visibility, single-trip time-temperature).

According to the two-dimensional kernel density analysis plot in Figure 13, the kernel density of bus single-trip time and visibility has a bipartite distribution, while the kernel density and temperature have a single kernel distribution.

After fusing bus single-trip time data, weather data (precipitation, visibility, temperature), and holiday data, the training dataset, test dataset, and prediction dataset are set up in the ratio of 6 : 1 : 1. Data pre-processing is done using python language, and data noise reduction is done using scipy's own filter. In this study, the one-way operation data of Beijing Bus No. 2 are used as an example for prediction.

#### 2.1.1. Model Evaluation Metrics

The root-mean-squared error (RMSE) and the mean absolute error (MAE) are the two most frequently used metrics to measure the accuracy of variables, and they are also two important yardsticks to evaluate models in machine learning. Therefore, these two indicators are selected to compare and analyze the prediction effects of different models in this paper. These two indicators mainly reflect the magnitude of error between the predicted and actual values, defined in Eq.

(3) $$\begin{array}{c} \text{RMSE}=\sqrt{\frac{1}{n}\sum {\left(y^{true}-y^{pred}\right)}^{2}}\\ \text{MAE}=\sqrt{\frac{1}{n}\sum \left|y^{true}-y^{pred}\right|}. \end{array}$$

In the above equation, the *y*^*true*^ denotes the actual value, the *y*^*pre*  *d*^ MAE reflects the error between the model prediction and the real value, and RMSE is more sensitive to the outliers, reflecting the model's stability. The smaller the RMSE and MAE values of the prediction results, the closer the predicted value and the actual value are, and the higher the prediction accuracy of the model [72]. The smaller the RMSE and MAE values are, the closer the predicted and actual values are, and the higher the prediction accuracy of the model.

#### 2.1.2. Experimental Parameter Settings

All algorithms are implemented using Python 3.7 running on a computer with a quad-core 2.6-GHz CPU and 16 GB random-access memory.

In terms of hyperparameter settings, based on factors such as error size, training efficiency, and degree of fit, a combination of grid search and cross-validation is used, and two timesteps of 4 and 6 are selected for training and prediction.

In the base model LR, fit_intercept is the default True. Since the dataset has been normalized, nomalize is false, and copy_X is set to True to avoid overwriting the original data. Set n_jobs to -1 to improve the operational efficiency. In the base model XGBoost, based on the amount of data, the number of iterations is set to 200, n_estimators = 200, and learning_rate = 0.4. The maximum depth of each tree Max_depth is 6, min_child_weight = 1, gamma is the default 0. In sampling, subsample = 1, because there are a few features, bytree, bylevel, and bynode are all 1. Because XGBoost has an advantage in training speed, so tree_method choose exact with higher precision. In the deep learning-based models LSTM and GRU, in order to prevent under-fitting or over-fitting, the grid search method is used to conduct multiple experiments to find the optimal epoch and batch_size. In LSTM, epoch = 200, batch_size = 16, neuron units = 4, dropout = 0.2. In GRU epoch = 150, batch_size = 4, neuron units = 4, dropout = 0.2. In KNN, since the distance weight needs to be considered, weight = 'distance', and the value of *k* is 4 according to the size of the data.

#### 2.1.3. Comparison of Prediction Results

In this study, two feature sets containing weather conditions and holidays and only weather conditions were selected for model training. The granularity of bus single-trip time used in this study is hour. The original data counts the bus single-trip time from 0:00 to 12:00 per hour, while the time granularity of weather data is 4 hours. In the case that time-step is one hour, this study adopts 4 and 6 as the experimental time-step, which can fully study the influence of weather on the bus single-trip time, and can also fully reflect the periodic regularity of the bus single-trip time. The experimental results obtained in these cases can compare and analyze the performance and generalization ability of the model from different perspectives.

**Figure 14:**
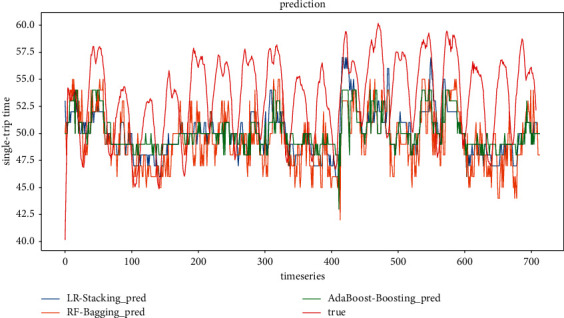
Ensemble model prediction results (timestep = 4).

**Figure 15:**
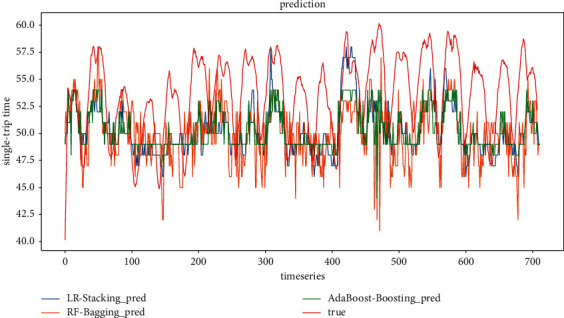
Ensemble model prediction results (timestep = 6).

*(1) Model evaluation considering weather conditions and holiday scenarios*. The comparison of the predicted and true values of the three ensemble models at step sizes of 4 and 6, respectively, is shown in Figures 14 and 15. It can be found that the prediction results of the ensemble model based on Random Forest are better than those of the other two ensemble models in both step sizes, and the fit is better and closer to the true value. The ensemble model based on AdaBoost has the second-best fit, and the prediction result of the ensemble model based on Linear Regression has the worst fit among the three ensemble models. In general, all three ensemble models can learn the fluctuation pattern of the real data, and the prediction result of AdaBoost has the least fluctuation compared with the other two ensemble models. It is not sensitive to the response of data peaks and troughs.

From the evaluation indices of the ensemble model in Table 2, both mae and rmse of RF-Bagging are the smallest, indicating that it has the smallest error, while *R*^2^ in the case of step size 4 is 0.909and in the case of step size 6 is 0.883; both are greater than 0.8, indicating that this model can learn the advantages of each sub-model and avoid its shortcomings, making the overall error lower and the fit higher, which is an excellent ensemble prediction model.

**Figure 16:**
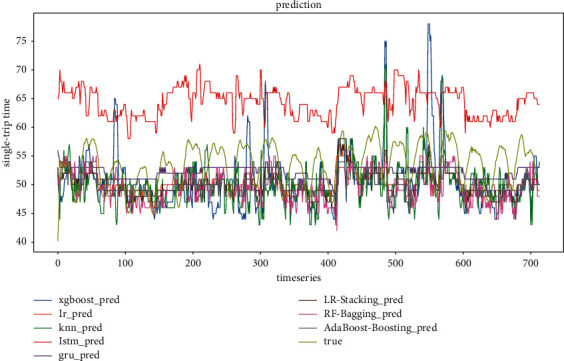
All model prediction results (timestep = 4).

**Figure 17:**
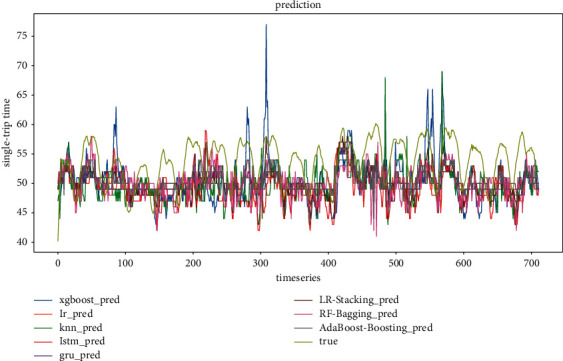
All model prediction results (timestep = 6).

Figures 16 and 17 show the predicted and actual values of the bus single-trip time derived from the training of five single models and three ensemble models. It can be seen that the single models, LSTM, LR, GRU, KNN, and XGBoost, all have inferior prediction results than the ensemble models. Among the five sub-models, the prediction results of LSTM have the largest deviation, and the prediction results of LR and GRU are the closest to the real values. This is mainly due to the dynamic nature of neural networks and the advantage of ensemble deep learning. In terms of model training time, LR, KNN, and XGBoost are much faster than LSTM and GRU and are more suitable for the short-time prediction of large data sets.

As shown in Table 3, among the sub-models, the training time of the LSTM model and the GRU model is quite different, mainly because LSTM and GRU require 200 and 150 rounds of learning, respectively, to achieve convergence. Among the five sub-models, LR completed the training and prediction in the shortest time and achieved the smallest error. In the ensemble model, RF-Bagging and LR-Stacking take much less time than AdaBoost-Boosting. On the whole, RF-Bagging obtains the most accurate prediction results in a shorter time.

From the evaluation indices of all models in Table 4, all three ensemble models are smaller than the sub-models in terms of error, which can fully illustrate the ensemble advantages of the ensemble models. In contrast, among the five sub-models, except for the LSTM with larger error, the errors of the remaining four models are relatively close, among which LR and GRU are better than KNN and XGBoost in terms of error. It is noteworthy that in the case of step size 4, the prediction bias of LSTM is larger in the case of timestep size 6, while the error decreases in the case of step size 6, indicating that the adjustment of step size optimizes the prediction accuracy of LSTM.

Table 5 shows the results of model testing. To verify the superiority of RF-Bagging, Wilcoxon signed-rank test and Friedman test were performed on all models in this study, and the test results are shown in Table 5. In the Wilcoxon signed-rank test, the rank of the absolute value of the difference between the observed value and the center position of the null hypothesis is added according to different signs as its test statistic. It is suitable for pairwise comparisons in *T*-tests, but does not require that the differences in paired data follow a normal distribution. This test only requires a symmetrical distribution, so it is more suitable for the comparative test of the predicted value of the bus single-trip time. Therefore, in order to verify the superiority of RF-Bagging, this study tested the predicted values of RF-Bagging with those of other seven models. Friedman test can take full advantage of all the information in the relevant sample. The prerequisites for using the Friedman test are (1) ordinal-level data, (2) three or more groups of data, and (3) randomly draw samples from the collocated values. Therefore, the Friedman test is also applicable to this study. From the test results, whether it is the Wilcoxon signed-rank test or the Friedman test, the *ρ* obtained is less than the significance level (0.05). This shows that RF-Bagging shows better prediction performance than other models when considering weather and holidays.

In summary, it can be seen that the three ensemble models have excellent prediction accuracy at both step sizes considering weather and holidays, and the ensemble model has a better fit than the sub-models. The Random Forest-based ensemble model constructed based on the Bagging ensemble idea has the best fit and prediction accuracy among the three ensemble models. This reflects the advantages of this model ensemble method: due to the use of random sampling, the variance of the trained model is small, and the generalization ability is strong; compared with the traditional decision tree model, it combines the results of multiple decision tree models, and the model ground effect will be better; in the case of large data fluctuations, the ensemble model based on Random Forest can better integrate the advantages of each sub-model and the prediction accuracy higher prediction accuracy. GRU and Linear Regression have higher approximation accuracy and generalization ability among the five sub-models, and the prediction results are closer to the real values than other sub-models.

**Figure 18:**
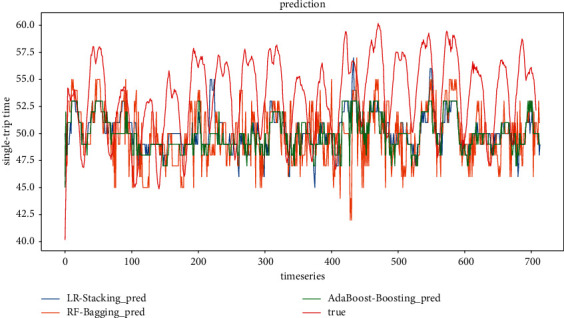
Ensemble model prediction results (timestep = 4).

**Figure 19:**
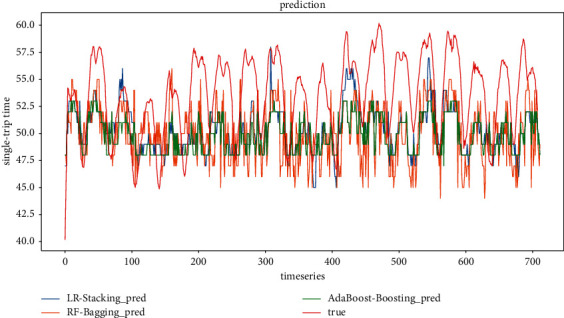
Ensemble model prediction results (timestep = 6).

*(2) Model evaluation considering only weather conditions scenarios*. The comparison between the predicted and true values of the three ensemble models for the scenarios considering only rainfall, visibility, and temperature with step sizes of 4 and 6, respectively, is shown in Figures 18 and 19. It can be found that, similar to the prediction results of the scenarios considering holidays and weather, the prediction results of the ensemble model constructed based on Random Forest at two-step sizes still outperform those of the other two ensemble models, which are closer to the true values. The prediction result of the ensemble model based on AdaBoost is the second best, and the prediction result of the ensemble model based on Linear Regression is the worst fit among the three ensemble models.

From the evaluation indices of the ensemble model in Table 6, the mae and rmse of RF-bagging are basically the smallest, but the error is not large with the other two ensemble models in this scenario, while *R*^2^ In the case of step size 4 is 0.836 and in step size 6 is 0.846; both are greater than 0.8. This indicates that this model can also learn the advantages of each sub-model under the condition of considering only the weather, which makes the overall error lower and the fit higher, and is a better prediction model in comparison.

**Figure 20:**
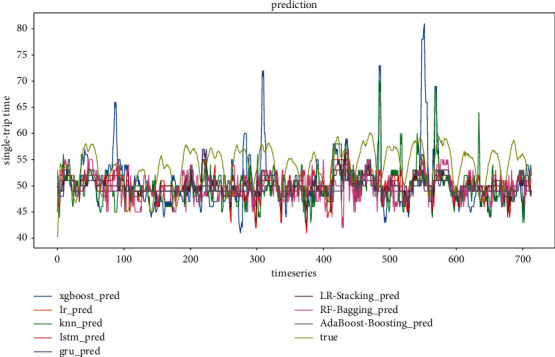
All model prediction results (timestep = 4).

**Figure 21:**
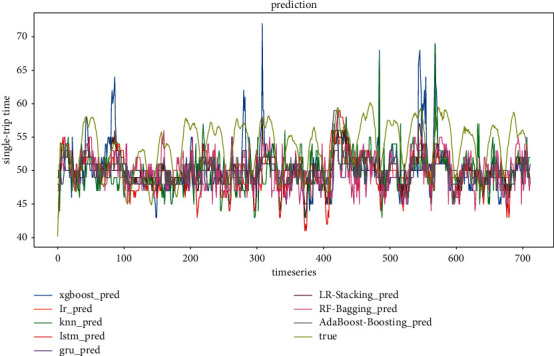
All model prediction results (timestep = 6).

Figures 20 and 21 show the predicted and actual values of bus single-trip time derived from the training of five single models and three ensemble models. It can be seen that LSTM, LR, GRU, KNN, and XGBoost are inferior to the ensemble models. It is worth noting that the prediction results of the XGBoost model in this context fluctuate more, indicating that the fit of XGBoost is not good in the case of feature reduction. In terms of model training time, LR, KNN, and XGBoost are also trained much faster than LSTM and GRU, which are more suitable for short-time prediction of large datasets.

As shown in Table 7, the training time and prediction time of the model are overall longer than when the weather and holidays are considered, mainly because of the increase in data features. As far as the base model is concerned, LR is still the model with the shortest training and prediction time. And the model with the smallest error becomes LSTM, which reflects the advantages of deep learning in the case of increasing data features. In terms of ensemble models, the training and prediction times of the three ensemble models are not significantly different.

As shown in Table 8, from the evaluation indices of all models, all three ensemble models are smaller than the sub-models in terms of error, fully illustrating that the ensemble models have the same advantages of model ensemble when only weather conditions are considered. Compared with the scenarios considering weather and holidays, among the five sub-models, the prediction error of LSTM at step size 4 is the smallest, while the prediction results of LR and LSTM are the closest to the true value, and the prediction results of GRU and LR at step size 6 are the closest to the true value. Combining the prediction results of the two scenarios illustrates that LR as a single prediction model has a more stable and high prediction accuracy when predicting bus single-trip time.

As shown in Table 9, the Wilcoxon signed-rank test and Friedman test results for models when only the weather is considered are shown in Table 8. It can be found that RF-Bagging also maintains its superiority in this scenario, and the *ρ* in the test results are all less than the significance level (0.05).

In conclusion, it can be seen that the three ensemble models have excellent prediction accuracy at both step sizes when only weather is considered, and the ensemble models have a better fit than the sub-models. In comparing the prediction results of the ensemble models, the ensemble model constructed based on Random Forest has higher prediction accuracy and fit, which further illustrates the applicability of the parallelization approach based on the idea of bagging ensemble in bus single-trip time prediction. By fitting different learners individually and training them simultaneously, an ensemble model is generated that is more robust than a single model. Meanwhile, Random Forest supports multiple tree ensemble, which can form a powerful heterogeneous ensemble algorithm to randomly select samples and features, reducing the effect of outliers and reducing overfitting.

## 3. Discussion

In this study, five single predictive models and three ensemble models are validated with real-world data in order to find the best method for bus single-trip time prediction. And the sensitivity of the prediction accuracy of each model to the number of features is also verified by adding an extra feature with data of holidays. The optimal model for predicting the bus single-trip time is evaluated by the two error evaluation indices of MAE and RMSE combined with the training and prediction efficiency of the model. In terms of a single prediction model, this study selected a linear regression model from the traditional statistical category. The simplest and easy-to-implement KNN and XGBoost that can effectively prevent overfitting are selected from the machine learning category. LSTM that can learn long-term dependency information and GRU that are more efficient are selected from the deep learning category. In terms of the ensemble method, this research selects RF-bagging, AdaBoosting, and LR-stacking from the three popular ensemble categories including bagging, boosting, and stacking. The purpose of this study is to compare the prediction accuracy, error, and efficiency of the ensemble predictive model and the single predictive model under different characteristics and timesteps and to find an optimal predictive model suitable for the bus single-trip time dataset.

In the case of verification part, the parameters of the model are adjusted by continuous trial and error, so that each model achieves a better balance in terms of prediction accuracy and training efficiency. Finally, the comparison between the predicted value and the real value of each model is obtained, and the MAE and RMSE of each model are calculated. Based on the result comparison between ensemble models and single prediction models, whether or not the holiday data are considered, the prediction accuracy of the ensemble models is higher than that of the single predictive models. This is mainly due to the fact that ensemble learning combines multiple base learners to obtain superior generalization ability compared to a single learner. This shows that the idea of ensemble learning is suitable for the prediction of bus single-trip time. For nonlinear time series data ensemble models that combine multiple weather conditions and holidays, the advantages of basic learners can be well combined. This experimental result also well confirms the current academic description of the advantages of ensemble learning.

Looking into the prediction results of the three ensemble models selected in this study, when there are many data features, the prediction error of the ensemble model is lower. In both cases, the prediction error of the RF-Bagging model constructed based on the bagging ensemble method is the lowest. This reflects the difference between ensemble methods of bagging and boosting: bagging focuses on reducing the variance of the model (preventing overfitting), while boosting focuses on reducing the skewness of the model (preventing underfitting). The nonlinear characteristics of the bus single-trip time data set used in this paper are relatively obvious since Beijing public transport has been affected by the COVID-19 pandemic and various social activities during the time period in which the data set is located. The ensemble idea of bagging is more suitable for such noisy datasets. While RF-bagging is based on a decision tree model which introduces random samples and attribute selection based on bagging ensemble. It makes RF-bagging less prone to overfitting in case of using high-noise datasets. The LR-Stacking constructed by stacking is dependent on basic learners. Once too many basic learners are unsuitable for processing noisy datasets, the accuracy of LR-Stacking will decrease.

On the other hand, from the prediction results of the five single predictive models, LR has the highest accuracy, while KNN and XGBoost of machine learning and LSTM and GRU of deep learning have comparable performance. This is also probably due to the dataset's high noise and nonlinear characteristics. Relatively complex machine learning and deep learning models are more seriously affected by data noise, and the degree of overfitting is higher than that of simpler LR.

From the perspective of algorithm complexity, among sub-models, LSTM and GRU, which belong to the deep learning category, are the most complex. In this study, LSTM and GRU have six layers of hidden nodes. The complexity of the two data dimensions is relatively high. They occupy most of the system resources and consume a long time for model training. However, the training and prediction time of other models is in the millisecond level, and the resource consumption is not high. Among them, LR has the fastest resource occupation and training speed. This is mainly due to the fact that LR only needs to store the eigenvalues of each dimension, so relatively speaking, the resource occupancy is small, and the calculation amount is only the number of features. Relevant, this study has fewer features, so the calculation speed is relatively fast. In ensemble models, LR-Stacking, which also uses LR as a secondary base learner, has the fastest resource occupancy and training speed. In the process of each iteration, Ada-Boosting gradually approaches the expected value from the two aspects of the detection rate and the misrecognition rate of positive samples to construct a cascaded classifier, which can only be achieved after iterative training generates a large number of weak classifiers, construction process. From this, it takes more time to train the classifier with a circular approximation, so its complexity is the highest among the three ensemble models.

The theoretical significance of this study is to verify the effectiveness of ensemble learning in the prediction of bus single-trip time. In ensemble learning, the RF-bagging model constructed by bagging is the most suitable for predicting the bus single-trip time. In addition, the RF-bagging model is versatile when dealing with nonlinear and noisy datasets, which can effectively prevent model overfitting. The practical significance of this study is that when the influence of many external factors makes the bus single-trip time become irregular, the ensemble model proposed in this research can provide the public transport managers and passengers with accurate bus single-trip time predictions. It provides convenience for passengers' travel and also provides a basis for managers to assist in decision-making. Based on the characteristics of the datasets used in this research, in future, more solutions will be proposed for excessive data noise.

## 4. Conclusion

Predicting traffic demand is a central issue in the organization of any transportation system, and the predictive demand could help to plan a reasonable supply in advance. From the perspective of public transportation, the distribution of bus single-trip time is needed in real time for travel planning, operation strategy formulation and adjustment, and contingency planning. This paper proposes a methodology for constructing a multi-model ensemble bus single-trip time prediction model based on the public transportation data, holiday data, and external weather data. The empirical analysis is conducted by using the proposed methodology and comparatively predict bus single-trip time based on the single models and the ensemble models. The specific research work done and research results obtained in this paper are mainly as follows:

1. The bus single-trip time data, holiday data, and external weather data are cleaned separately, including data redundancy, data gap filling, and abnormal data processing and noise reduction. Moreover, the cleaned datasets are fused with multiple sources to provide data support for the further bus single-trip time prediction model building.
2. A data-driven bus single-trip time prediction framework is constructed, including three steps of data analysis, feature extraction, and prediction modeling.
3. Three ensemble models of bagging (Random Forest), boosting (AdaBoost), and stacking (Linear Regression) were constructed for predicting bus single-trip time based on three-model ensemble methods.
4. A case analysis was conducted using real data of Beijing bus Line No. 2. The advantages and disadvantages of the five base models and the three ensemble models were compared and analyzed. The ensemble model for bus single-trip time prediction is constructed, and the constructed ensemble model is used to make short-time predictions of bus single-trip time under normalization. The real values are used as the baseline for detailed comparison with the prediction results of the constructed model from the perspective of the single model and the ensemble model. The results of case analysis show the following: (1) in general, the prediction results of the ensemble model are commonly better than those of the sub-models, regardless of whether the nonlinear time-series data are volatile or regular, which reflects the strong benefits of ensemble learning; (2) the ensemble model of Random Forest built by the method of bagging ensemble the advantages and disadvantages of the five sub-models. The overall prediction results are smoother and better than those of the five sub-models and is closer to the real value. Since the ensemble model fully learns the laws between the independent variables and the prediction results of the sub-models instead of simply integrating the prediction results directly. The overall prediction effect is better, which brings out the optimal solution for the prediction model in each scenario. (3) Among the single predictive model, LR is the best model with high prediction accuracy but with not high computational cost that is also easy to implement. It can be applied to distributed data and handle large data with fewer resources. In addition, LR is robust to a small noise in the data and does not suffer from slight multicollinearity, making it an optimal solution for a single prediction model.

Further research on the bus single-trip time prediction problem can be done in the following two areas:

1. The selection of sub-models and the number of selecting rounds mainly rely on historical experience, and the subsequent optimization algorithm can be considered to make the selection more intelligent and reasonable.
2. This paper only predicts the bus single-trip time from a theoretical point of view and provides data support for the subsequent development of emergency strategies. The subsequent development of emergency plans, the arrangement of travel plans, and the practical application of bus connections still need further discussion with relevant staff in the field.

## Figures and Tables

**Algorithm 1:**
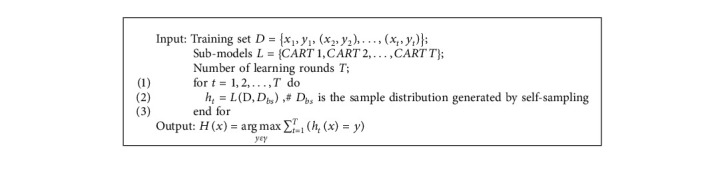
RF-bagging ensemble model.

**Algorithm 2:**
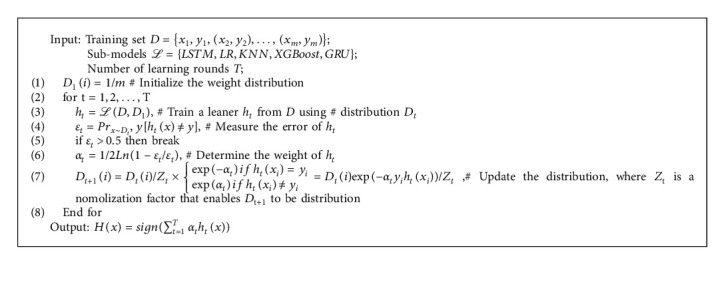
AdaBoost-Boosting ensemble model.

**Algorithm 3:**
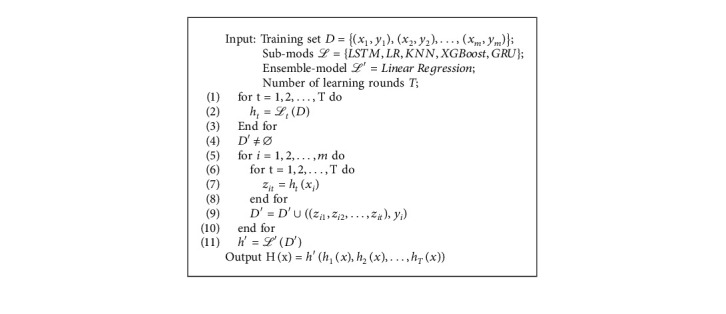
LR-Stacking ensemble model.

**Table 1:** Comparison of advantages and disadvantages of popular models.

Category	Commonly used models	Advantages	Disadvantages	References
History of the same period	Smoothing method	Easy understandability, better results in normal conditions and with large time granularity	Excessive reliance on data patterns from historical data	Omkar and Kumar [32]

Time series	Kalman filtering	Applicable to time series data and interpretability	Unsuitable for capturing nonlinear data patterns	Zhou et al. [33]
	AR(Auto regressive)			Li et al. [34]
	ARIMA			Gummadi and Edara [35]

Machine learning	SVM SVR	Suitable for learning nonlinear features in data	Low computational efficiency at high data volumes	Li and Xu [36]
	K-nearest neighbor			Sun et al. [37]
	Linear regression			Khiari and Olaverri-Monreal [38]
	Decision tree			Alajali et al. [39]
	Random forest			Zhou et al. [40]

Deep learning	RNN	Applicable for learning linear and nonlinear patterns with good data fitting capability	Low interpretability and low efficiency	Pang et al. [41]
	LSTM			Agafonov and Yumaganov [23, 42]
	GRU			Shu et al. [43]

Ensembled model	AdaBoost	Applicable to select the appropriate base model for ensemble according to the characteristics of different datasets	Prone to overfitting, low interpretability, and poor results when data are unbalanced	Zhou et al. [44]
	Bootstrapped aggregation			Vaish et al. [45]
	Stacked generalization			Sharma et al. [46]
	Gradient boosting Machines, GBM			Monego et al. [47, 48]
	Gradient boosted regression Trees, GBRT			Chen et al. [49]

Combined model	Direct averaging, weighted averaging, and other combinations	High applicability with various sub-models and combinations	Subjective on choosing the combination method and sub-models	Yan et al. [50]

**Table 2:** Model evaluation indices.

Ensemble models	TimeStep = 4			TimeStep = 6		
	MAE	RMSE	**R** ^2^	MAE	RMSE	**R** ^2^
LR-stacking	4.317347	5.236942	0.155475	4.062111	4.808697	0.196314
RF-bagging	3.232055	4.313371	0.909258	3.895147	4.671306	0.883880
AdaBoost-boosting	4.086188	5.040191	0.489591	3.973029	4.724360	0.404368

**Table 3:** Model training and prediction time.

Models	TimeStep = 4		TimeStep = 6	
	Training	Prediction (ms)	Training	Prediction (ms)
LSTM	115 min	1257	121 min	1471
GRU	119 min	727	126 min	1103
LR	7 ms	2	16 ms	3
KNN	77 ms	63	98 ms	82
XGBoost	3619 ms	48	5192 ms	73
LR-stacking	58 ms	11	52 ms	56
RF-bagging	329 ms	79	417 ms	136
AdaBoost-boosting	2351 ms	92	3162 ms	141

**Table 4:** Model evaluation indices.

Models	TimeStep = 4		TimeStep = 6	
	MAE	RMSE	MAE	RMSE
LSTM	11.130382	11.977166	5.008087	5.851334
GRU	4.696761	5.694506	4.307110	5.269999
LR	4.324053	5.275761	4.038138	4.842730
KNN	4.931067	6.001118	4.690769	5.538258
XGBoost	5.112036	6.396894	4.604660	5.554114
LR-stacking	4.317347	5.236942	4.062111	4.808697
RF-bagging	3.232055	4.313371	3.895147	4.671306
AdaBoost-boosting	4.086188	5.040191	3.973029	4.724360

**Table 5:** Wilcoxon Single-Rank and Friedman test result.

Models	TimeStep = 4		TimeStep = 6	
	Wilcoxon single-rank test (*ρ* ≤ 0.05)	Friedman test (*ρ* ≤ 0.05)	Wilcoxon single-rank test (*ρ* ≤ 0.05)	Friedman test (*ρ* ≤ 0.05)
LSTM	0.00004	0.000298	0.00251	0.000463
GRU	0.00891		0.00830	
LR	0.00916		0.00962	
KNN	0.00612		0.00973	
XGBoost	0.00315		0.00988	
LR-stacking	0.00979		0.01374	
AdaBoost-boosting	0.01693		0.01421	

**Table 6:** Model evaluation indices.

Ensemble model	TimeStep = 4			TimeStep = 6		
	MAE	RMSE	**R** ^2^	MAE	RMSE	**R** ^2^
LR-stacking	4.129265	5.092021	0.123339	4.098628	4.870725	0.205406
RF-bagging	4.146337	5.069491	0.836024	3.935297	4.683990	0.846075
AdaBoost-boosting	4.347092	5.273396	0.412592	4.139669	4.920567	0.390879

**Table 7:** Model training and prediction time.

Models	TimeStep = 4		TimeStep = 6	
	Training	Prediction (ms)	Training	Prediction (ms)
LSTM	85 min	986	106 min	1326
GRU	93 min	645	114 min	735
LR	4 ms	1	6 ms	2
KNN	64 ms	52	71 ms	68
XGBoost	2639 ms	32	3283 ms	42
LR-stacking	21 ms	6	35 ms	21
RF-bagging	214 ms	51	257 ms	82
AdaBoost-boosting	1427 ms	72	1974 ms	102

**Table 8:** Model evaluation indices.

Models	TimeStep = 4		TimeStep = 6	
	MAE	RMSE	MAE	RMSE
LSTM	4.306787	5.199513	4.481952	5.300563
GRU	4.861043	5.786797	4.392949	5.170590
LR	4.539661	5.596035	4.361890	5.276523
KNN	4.954903	6.027883	4.684654	5.524954
XGBoost	5.214021	6.529260	4.698066	5.579520
LR-stacking	4.129265	5.092021	4.098628	4.870725
RF-bagging	4.146337	5.069491	3.935297	4.683990
AdaBoost-boosting	4.347092	5.273396	4.139669	4.920567

**Table 9:** Wilcoxon Single-Rank and Friedman test results.

Models	TimeStep = 4		TimeStep = 6	
	Wilcoxon single-rank test (*ρ* ≤ 0.05)	Friedman test (*ρ* ≤ 0.05)	Wilcoxon single-rank test (*ρ* ≤ 0.05)	Friedman test (*ρ* ≤ 0.05)
LSTM	0.00942	0.000327	0.00887	0.000409
GRU	0.00725		0.00891	
LR	0.00932		0.00736	
KNN	0.00462		0.00623	
XGBoost	0.00152		0.00693	
LR-stacking	0.00979		0.01729	
AdaBoost-boosting	0.00957		0.00932	

## Data Availability

The bus single-trip time data used to support the findings of this study may be released upon application to the Beijing Transportation Operations Coordination Center (TOCC), who can be contacted at http://jtw.beijing.gov.cn/. Holiday data can be obtained at http://www.gov.cn/shuju/index.htm. Weather data can be obtained at https://www.wunderground.com/history/.

## References

[1] Kumar B. A., Vanajakshi L., Subramanian S. C. (2017). Pattern-based time-discretized method for bus travel time prediction. *Journal of Transportation Engineering, Part A: Systems*.

[2] Subhe D (2022). Network-scale traffic modeling and forecasting with graphical lasso and neural networks. *Journal of Transportation Engineering*.

[3] Chen G., Yang X., An J., Zhang D. (2012). Bus-arrival-time prediction models: link-based and section-based. *Journal of Transportation Engineering*.

[4] Jianhui L., Yang S., Zhenhao S., Zhu M. (2012). A method of road traffic flow prediction based on ARIMA model. *Proceedings of the Aeit 2012: 2012 2nd International Conference on Aerospace Engineering and Information Technology Hong Kong Education Soc: Tseung Kwan O*.

[5] Chikkakrishna N. K., Hardik C., Deepika K., Sparsha N. (2019). SHORT-TERM TRAFFIC PREDICTION USING SARIMA AND FbPROPHET. *Proceedings of the 2019 Ieee 16th India Council International Conference (Ieee Indicon 2019)*.

[6] Mao S., Xiao X., Gao M., Wang X., He Q. (2018). Nonlinear fractional order Grey model of urban traffic flow short-term prediction. *Journal of Grey System*.

[7] Kalman A. (2022). *Filter Approach to Dynamic OD Flow Estimation for Urban Road Networks Using Multi-Sensor Data - Lu - 2015 - Journal of Advanced Transportation*.

[8] Pan X., Zhou W., Lu Y., Sun N. (2019). Prediction of network traffic of smart cities based on DE-BP neural network. *IEEE Access*.

[9] Satrinia D., Saptawati G. A. P. Traffic speed prediction from GPS data of taxi trip using support vector regression.

[10] Parida M., Kumar K., Katiyar V. K. (2014). Prediction of urban traffic noise using artificial neural network approach. *Environ. Eng. Manag. J.*.

[11] Yanqiu W., Qiang L., Jian Z., Lifeng M., Yu W. The city traffic flow prediction based on BP neural network.

[12] Ban X., Guo C., Li G., Cao J., Mao K., Wu J., Lendasse A. (2016). Application of Extreme learning machine on large scale traffic congestion prediction. *Proceedings of the Proceedings of Elm-2015, Vol 1: Theory, Algorithms and Applications*.

[13] Yu H., Xiao R., Du Y., He Z. A bus-arrival time prediction model based on historical traffic patterns.

[14] Pili F., Olivo A., Barabino B. (2019). Evaluating alternative methods to estimate bus running times by archived automatic vehicle location data. *IET Intelligent Transport Systems*.

[15] Crudden S. O., Berrebi S. (2018). An open-source framework to implement kalman filter bus arrival predictions. *Networks and Spatial Economics*.

[16] Tang Z. P., Chen Z. X., Sun J. P., Hu Y. T., Zhao M. (2019). Noise prediction of traction gear in high-speed electric multiple unit. *International Journal of Simulation Modelling*.

[17] Mousavian S., Valenzuela J., Wang J. (2013). Real-time data reassurance in electrical power systems based on artificial neural networks. *Electric Power Systems Research*.

[18] Wang X., Huang L., Huang H., Li B., Xia Z., Li J. (2020). An ensemble learning model for short-term passenger flow prediction. *Complexity*.

[19] Zhong G., Yin T., Li L., Zhang J., Zhang H., Ran B. (2022). Bus travel time prediction based on ensemble learning methods. *IEEE Intelligent Transportation Systems Magazine*.

[20] Balasubramanian P., Rao R. (2015). An adaptive long-term bus arrival time prediction model with cyclic variations. *Journal of Public Transportation*.

[21] Ghosh B., Basu B., O’Mahony M. (2009). Multivariate short-term traffic flow forecasting using time-series analysis. *IEEE Transactions on Intelligent Transportation Systems*.

[22] Gu W., Gayah V. V., Cassidy M. J., Saade N. (2014). On the impacts of bus stops near signalized intersections: models of car and bus delays. *Transportation Research Part B: Methodological*.

[23] Agafonov A., Yumaganov A., Lu H., Tang H., Wang Z. (2019). Bus arrival time prediction with LSTM neural network. *Proceedings of the Advances in Neural Networks - Isnn 2019, Pt I*.

[24] Yu B., Lam W. H. K., Tam M. L. (2011). Bus arrival time prediction at bus stop with multiple routes. *Transportation Research Part C: Emerging Technologies*.

[25] Bie Y., Wang D., Qi H. (2012). Prediction model of bus arrival time at signalized intersection using GPS data. *Journal of Transportation Engineering*.

[26] Dhivya Bharathi B., Anil Kumar B., Achar A., Vanajakshi L. (2020). Bus travel time prediction: a log-normal auto-regressive (ar) modelling approach. *Transportmetrica: Transportation Science*.

[27] Chang H., Park D., Lee S., Lee H., Baek S. (2010). Dynamic multi-interval bus travel time prediction using bus transit data. *Transportmetrica*.

[28] Liu J., Xiao G., Shen W. M., Paredes H., Luo J., Barthes J. P. (2019). Efficient bus arrival time prediction based on spark streaming platform. *Proceedings of the Proceedings of the 2019 Ieee 23rd International Conference on Computer Supported Cooperative Work in Design (Cscwd)*.

[29] Hua X., Wang W., Wang Y., Ren M. (2017). Bus arrival time prediction using mixed multi-route arrival time data at previous stop. *Transport*.

[30] Wu S., Nagahashi H. (2014). Parameterized AdaBoost: introducing a parameter to speed up the training of real AdaBoost. *IEEE Signal Processing Letters*.

[31] Kotsiantis S. (2011). Combining bagging, boosting, rotation forest and random subspace methods. *Artificial Intelligence Review*.

[32] Omkar G., Kumar S. V., Singh U. P., Chahar B. R., Yadav H. R. P., Vij S. K. (2018). Development of simple exponential smoothing model for traffic flow prediction under heterogeneous traffic conditions. *Proceedings of the Urbanization Challenges in Emerging Economies: Energy and Water Infrastructure; Transportation Infrastructure; and Planning and Financing*.

[33] Zhou T., Jiang D., Lin Z., Han G., Xu X., Qin J. (2019). Hybrid dual kalman filtering model for short-term traffic flow forecasting. *IET Intelligent Transport Systems*.

[34] Li Z., Yan H., Zhang C., Tsung F. (2020). Long-short term spatiotemporal tensor prediction for passenger flow profile. *IEEE Robotics and Automation Letters*.

[35] Gummadi R., Edara S. R., Satapathy S. C., Tavares J., Bhateja V., Mohanty J. R. (2018). Analysis of passenger flow prediction of transit buses along a route based on time series. *Proceedings of the Information and Decision Sciences*.

[36] Li C., Xu P. (2021). Application on traffic flow prediction of machine learning in intelligent transportation. *Neural Computing & Applications*.

[37] Sun B., Cheng W., Goswami P., Bai G. (2018). Short-term traffic forecasting using self-adjusting k-nearest neighbours. *IET Intelligent Transport Systems*.

[38] Khiari J., Olaverri-Monreal C., DiFatta G., Sheng V., Cuzzocrea A., Zaniolo C., Wu X. (2020). Boosting algorithms for delivery time prediction in transportation logistics. *Proceedings of the 20th Ieee International Conference on Data Mining Workshops (Icdmw 2020)*.

[39] Alajali W., Zhou W., Wen S., Wang Y. (2018). Intersection traffic prediction using decision tree models. *Symmetry*.

[40] Zhou X., Lu P., Zheng Z., Tolliver D., Keramati A. (2020). Accident prediction accuracy assessment for highway-rail grade crossings using random forest algorithm compared with decision tree. *Reliability Engineering & System Safety*.

[41] Pang J., Huang J., Du Y., Yu H., Huang Q., Yin B. (2019). Learning to predict bus arrival time from heterogeneous measurements via recurrent neural network. *IEEE Transactions on Intelligent Transportation Systems*.

[42] Ren J. F., Ye C. M., Yang F. (2020). A novel solution to jsps based on long short-term memory and policy gradient algorithm. *International Journal of Simulation Modelling*.

[43] Shu W., Cai K., Xiong N. N. (2021). A short-term traffic flow prediction model based on an improved gate recurrent unit neural network. *IEEE Transactions on Intelligent Transportation Systems*.

[44] Zhou T., Han G., Xu X. (2017). *δ* -agree AdaBoost stacked autoencoder for short-term traffic flow forecasting. *Neurocomputing*.

[45] Vaish J., Datta S. S., Seethalekshmi K. Short-term load forecasting using bootstrap aggregation based ensemble method.

[46] Sharma S. R., Singh B., Kaur M. (2022). A novel approach of ensemble methods using the stacked generalization for high-dimensional datasets. *IETE Journal of Research*.

[47] Monego V. S., Anochi J. A., de Campos Velho H. F. (2022). South America seasonal precipitation prediction by gradient-boosting machine-learning approach. *Atmosphere*.

[48] Tampubolon P., Girsang A. S. (2021). Classification of attacks through the type of protocol using data mining. *JSMS*.

[49] Chen Y., Lv Y., Ye P., Zhu F. Traffic-condition-awareness ensemble learning for traffic flow prediction.

[50] Yan P., Zhang L., Feng Z., Zhang J. Research on logistics demand forecast of port based on combined model.

[51] Billings D., Yang J.-S. Application of the ARIMA models to urban roadway travel time prediction - a case study.

[52] Rapant L., Montemanni R. (2019). Traffic speed prediction using ensemble kalman filter and differential evolution. *Proceedings of the 2018 6th International Conference on Traffic and Logistic Engineering (Ictle 2018) E D P Sciences: Cedex A*.

[53] He P., Jiang G., Lam S.-K., Tang D. (2019). Travel-time prediction of bus journey with multiple bus trips. *IEEE Transactions on Intelligent Transportation Systems*.

[54] Bai C., Peng Z.-R., Lu Q.-C., Sun J. (2015). Dynamic bus travel time prediction models on road with multiple bus routes. *Computational Intelligence and Neuroscience*.

[55] Bidwai S., Wali U., Patil S. (2021). A comparative study of Markov chain and deep learning predictive models in spectrum sensing. *JSMS*.

[56] Vennelakanti A., Shreya S., Rajendran R., Sarkar D., Muddegowda D., Hanagal P. Traffic sign detection and recognition using a CNN ensemble. *Proceedings of the 2019 Ieee International Conference on Consumer Electronics (Icce)*.

[57] Xiao J., Xiao Z., Wang D., Bai J., Havyarimana V., Zeng F. (2019). Short-term traffic volume prediction by ensemble learning in concept drifting environments. *Knowledge-Based Systems*.

[58] Xiao J. (2019). SVM and KNN ensemble learning for traffic incident detection. *Physica A: Statistical Mechanics and Its Applications*.

[59] Guo Y., Wang X., Xiao P., Xu X. (2020). An ensemble learning framework for convolutional neural network based on multiple classifiers. *Soft Computing*.

[60] Tang B., Chen Q., Wang X., Wang X., Wong K. W., Mendis B. S. U., Bouzerdoum A. (2010). Reranking for stacking ensemble learning. *Proceedings of the Neural Information Processing: Theory and Algorithms, Pt I*.

[61] Jiang H., Zheng W., Luo L., Dong Y. (2019). A two-stage minimax concave penalty based method in pruned AdaBoost ensemble. *Applied Soft Computing*.

[62] Huang H., Huang L., Jiao F., Song R., Li J. Data-driven prediction of one-way bus running time: an integrated model.

[63] Yu B., Wang H., Shan W., Yao B. (2018). Prediction of bus travel time using random forests based on near neighbors. *Computer-Aided Civil and Infrastructure Engineering*.

[64] Feng Z., Mo L., Li M. A random forest-based ensemble method for activity recognition.

[65] Rooney N., Patterson D., Nugent C. (2007). Non-strict heterogeneous stacking. *Pattern Recognition Letters*.

[66] Liao Z., Su M., Ning G., Liu Y., Wang T., Zhou J. (2021). A novel stacked generalization ensemble-based hybrid PSVM-PMLP-MLR model for energy consumption prediction of copper foil electrolytic preparation. *IEEE Access*.

[67] Shi S., Liu Y., Huang Y., Zhu S., Liu Y., Guo M. Z., Zhao L., Wang L. P. (2008). Active learning for KNN based on bagging features. *Proceedings of the Icnc 2008: Fourth International Conference on Natural Computation, Vol 7*.

[68] Tang J., Zheng L., Han C., Liu F., Cai J. (2020). Traffic incident clearance time prediction and influencing factor Analysis using Extreme gradient boosting model. *Journal of Advanced Transportation*.

[69] Kim Y., Soh W. (2021). A study on character recognition of Korean vehicle license plates based on deep learning. *JSMS*.

[70] Fu R., Zhang Z., Li L. Using LSTM and GRU neural network methods for traffic flow prediction.

[71] Wu P. J., Yang D. (2021). E-commerce workshop scheduling based on deep learning and genetic algorithm. *International Journal of Simulation Modelling*.

[72] Willmott C. J., Matsuura K. (2005). Advantages of the mean absolute error (MAE) over the root mean square error (RMSE) in assessing average model performance. *Climate Research*.

